# Giant Scalp Melanoma: A Case Report and Review of the Literature

**Published:** 2012-11-16

**Authors:** Jessica A. Ching, Lisa Gould

**Affiliations:** ^a^Department of Surgery; ^b^James A. Haley Veterans’ Hospital, University of South Florida, Tampa

## Abstract

**Introduction:** Among malignant melanoma lesions, those occurring on the scalp and neck have a particularly poor prognosis. In this case report, we present the largest melanoma of the head and neck and one of the largest melanomas of any anatomic site reported in the literature to date.

**Methods:** The biopsy revealed left scalp melanoma with a Breslow's thickness of at least 14 mm, and final needle aspiration of lymphadenopathy was consistent with malignant melanoma. Preoperative staging was T4aN3Mx. Wide local excision with 3-cm margins was performed, which included excision of the left ear en-bloc, along with a selective left neck dissection. Reconstruction was performed with a Bilayer Wound Matrix (Integra, 311 Enterprise Drive, Plainsboro, New Jersey) and eventual thin split-thickness skin graft.

**Results:** Final pathology of the left scalp en-bloc excision was a 14.5 × 10.4 cm malignant melanoma, Breslow's thickness of 18 mm. Numerous lymph nodes were positive for melanoma as well. Final pathologic staging was determined to be T4b N3 M1, Stage IV. Later the patient underwent split-thickness skin graft placement on the left scalp acellular dermal matrix, which healed with complete graft take.

**Discussion:** This case report demonstrates a unique presentation of a giant melanoma. With few other cases reported for comparison, it appears our patient's prognosis is poor, despite treatment according to current guidelines.

Among malignant melanoma lesions, those occurring on the scalp and neck have a particularly poor prognosis, accounting for 6% of all melanomas but 10% of all melanoma-related deaths according to one study.^1^ According to recent literature, patients with scalp or neck melanomas died 1.84 times more frequently than those with extremity melanomas, and these patients have up to a mortality 3 times patients with melanoma of the face as well.^1^^,^^2^ Compared to other sites, scalp melanomas are more likely to appear in older male patients, and upon presentation to be thicker, ulcerated, lentigo maligna melanoma or nodular melanoma, and have metastasized to regional lymph nodes.^1^ In this case report, we present the largest scalp melanoma to the author's knowledge and one of the largest melanomas of any anatomic site reported in the literature to date.

## METHODS

**Figure 1:**
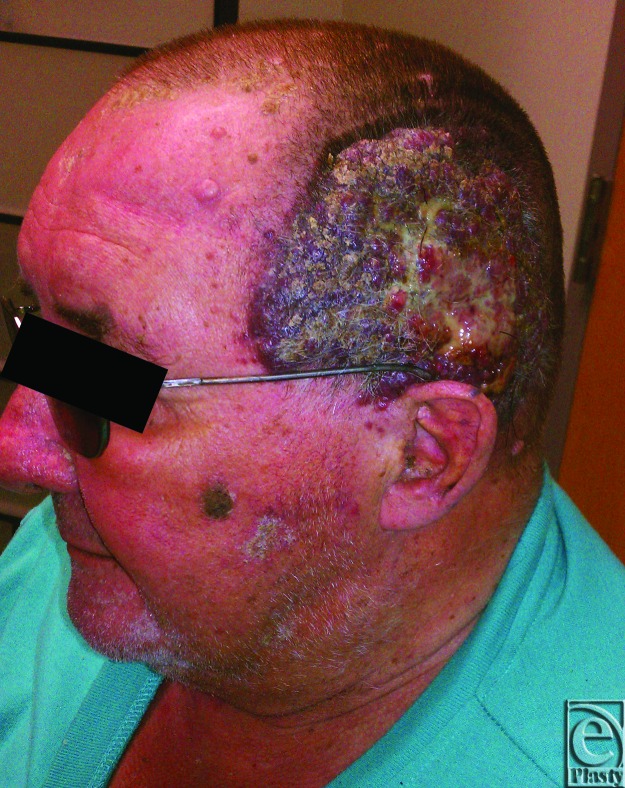
The left scalp lesion upon initial presentation to our clinic.

Our patient is a 70-year-old gentleman who initially presented for evaluation of a left scalp lesion (Fig 1). The lesion was present for 3 months, during which he had noted it increasing in size. Per patient report, it originally began as a small raised lesion above the left ear, which developed a dark streak heading posteriorly, and finally resulted in a 10×13 cm exophytic mass with irregular dark borders and scattered areas of ulceration. There was palpable lymphadenopathy, notably a left upper level 5 posterior cervical node of 3 cm in diameter and a left level 2 node of 4 cm in diameter. Biopsies from this first visit were consistent with nodular invasive malignant melanoma, Clark level V, Breslow's thickness of at least 14 mm. A subsequent final needle aspiration of the enlarged lymph nodes was performed, revealing metastatic malignant melanoma. Computed tomographic scan showed invasion of the left parotid gland and cartilage of the left ear; however, there was no obvious cranial involvement by the lesion. A full workup for metastatic disease was completed, including computed tomography of the thorax, abdomen, and pelvis, which did not show any suspicious lesions. After discussion at our institutional Tumor Board, the patient was preoperatively staged as T4aN3Mx. The patient inclined to pursue surgical resection of the lesion and selective left neck dissection.

Wide local excision with 3-cm margins was performed of the left scalp malignant melanoma, which included excision of the left ear en-bloc. There was a second lesion of the right scalp of 1×1 cm suspicious for malignant melanoma noted prior to surgery. An excisional biopsy was performed for the right scalp lesion and closed primarily during the same procedure. After excision of the scalp lesions was completed, a selective left neck dissection was performed. With the patient's medical comorbidities and extent of disease, he was not a candidate for large flap reconstruction. Reconstruction with a Bilayer Wound Matrix (Integra, 311 Enterprise Drive, Plainsboro, New Jersey) was chosen to allow complete evaluation of the excisional margins of the scalp lesions prior to final closure of the defect with a thin split-thickness skin graft.

## RESULTS

**Figure 2:**
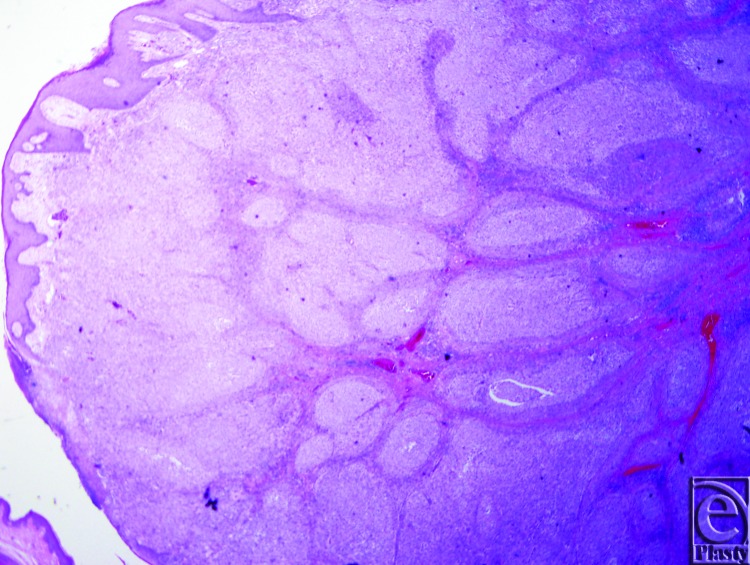
The lesion at 20× magnification.

**Figure 3:**
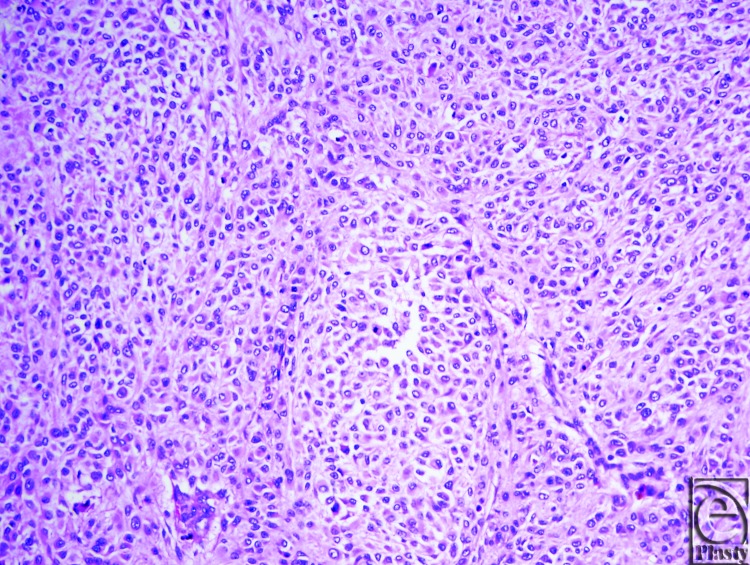
The lesion at 200× magnification.

Final pathology of the left scalp en-bloc excision revealed a 14.5 × 10.4 cm malignant melanoma, Clark level V, Breslow's thickness of 18 mm, mitotic rate 35/mm^2^ (Figs 2 and 3). In addition, the right scalp lesion was a separate primary malignant melanoma. The neck dissection yielded 20 lymph nodes involved by metastatic melanoma out of 55 total lymph nodes examined. The anterior-inferior margin of the left scalp melanoma was involved; however, the patient declined to proceed with further resection. Final pathologic staging was determined to be T4b N3 M1, Stage IV.

**Figure 4:**
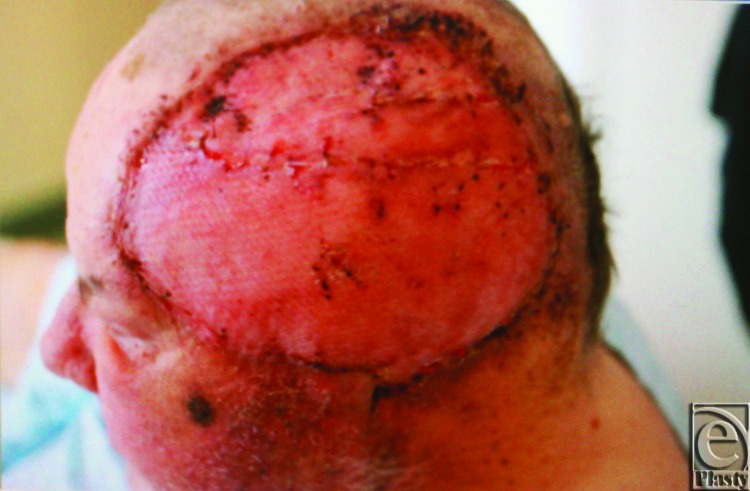
Our patient with healed split-thickness skin graft over the resection defect.

Four weeks later, the patient was returned to the operating room for split-thickness skin graft placement on the left scalp acellular dermal matrix. The patient tolerated the procedure well, and the skin graft healed well with complete graft take (Fig 4).

## DISCUSSION

To the authors’ knowledge, this is the largest diameter melanoma of the head and neck reported in the literature, and also one of the largest melanomas reported of any anatomic site. Giant melanomas are greater than 10 cm in diameter, and thick melanomas are those with a Breslow's depth of greater than 4 mm.^3^ The limited reported cases of giant melanomas of all body sites were reviewed and their data compiled (Table 1).

Presentation of a giant melanoma has been documented of the scalp,^4^ arm,^5^^,^^6^ abdomen,^7^ and back.^3^^,^^8^^,^^9^ The diameter of a giant malignant melanoma has been reported up to 20 cm,^5^ with Breslow's depth of 0.45 mm to 100 mm (mean of 48.6 mm). In our case, the giant melanoma was 18 mm in Breslow's depth and 14.5 cm in maximum diameter, which is the largest diameter reported for a scalp melanoma. The majority of the cases presented with clinically palpable regional lymphadenopathy, approximately 83% of cases where the lymph node status was reported (n = 6). To the authors’ knowledge, only one case reported in the literature did not present with metastatic disease (n = 7). The growth of the lesion prior to diagnosis ranges from 6 months to 15 years; however, in our patient the giant melanoma grew rapidly, only requiring 3 months to reach its massive size.

Despite the rare presentation of a rapidly enlarging mass of significant size, this does not alter the treatment algorithm. On the basis of current literature, the melanoma treatment guidelines published by the National Comprehensive Cancer Network for those melanomas with Breslow depth of 4 mm and greater apply to these giant lesions as well.^10^ Melanomas with depth of 4 mm or more are classified as T4 lesions with staging from IIc to IV, depending on nodal involvement and metastatic disease.^10^ Our patient did not have brain metastasis, but in advanced disease brain metastasis is common. Patients with stage IV disease have been documented to develop brain metastases up to 37% of the time.^11^ Only one case found in our review of the literature could be equivalent to a stage IIc; other cases would be at least stage III due to clinically evident lymphadenopathy. According to the compiled data, the likelihood of regional and metastatic disease is high with giant, thick melanomas, often requiring wide local excision in combination with significant lymph node dissections per the National Comprehensive Cancer Network guidelines.^10^ Inadequate margins or multiple lymph node involvement, as in our case, are indications for adjuvant radiation therapy, and consideration of adjuvant interferon alpha is also warranted.^10^ With nodal involvement, at least stage III, 5-year survival ranges from 20% to 70%, which is roughly half of the survival rates when there is no nodal involvement.^10^ Overall, metastatic disease on presentation is consistent with a 10% overall survival.^10^ When comparing our case to the few others reported of giant melanoma, it appears our patient's prognosis is poor, despite receiving treatment according to current guidelines.

## Figures and Tables

**Table 1:** Summary data for all case reports of giant melanomas found by the authors in a review of the literature

Case	Patient	Location	Length of Growth	Surface Area, cm	Breslow's Depth, mm	Regional LAD	Metastatic Disease	Treatment
Our current case	70 yo M	Scalp	3 mo	14.5 × 10.4	18	Yes	Yes	WLE
								SND
Panajotovic^4^	57 yo M	Scalp	3 y	12 × 10	100	No	No	WLE
Grisham^3^	45 yo F	Back	>1 y	13 x NR	55	Yes	Nr	WLE
								Ax ND
Eisen^8^	47 yo M	Back	6 mo	8 × 9	40	NR	Yes	WLE
Kruijff^9^	56 yo F	Back	NR	8 × 6	48	Yes	Yes	BLE
								Bil Ax ND
Tseng patient A^6^	88 yo M	Arm	NR	8 × 8	31	Yes	Yes	WLE
								Ax ND
Tseng patient B^6^	63 yo M	Arm	>1 y	19 × 19	75	Yes	Yes	WLE
								Ax ND
De Giorgi^7^	45 yo F	Abdomen	15 y	16 x NR	0.45	NR	NR	NR
Del Boz^5^	29 yo F	Arm	8 mo	20 × 20	70	NR	Yes	NR

AxND indicates complete axillary node dissection; Bil Ax ND, bilateral complete axillary node dissection; yo, year-old; LAD, lymphadenopathy; M, male; NR, not reported; SND, selective neck dissection; WLE, wide local excision.

## References

[1] Lachiewicz AM, Berwick M, Wiggins CL, Thomas NE (2008). Survival differences between patients with scalp or neck melanoma and those with melanoma of other sites in the Surveillance, Epidemiology, and End Results (SEER) program. Arch Dermatol.

[2] Leong SP, Accortt NA, Essner R (2006). Impact of sentinel node status and other risk factors on the clinical outcome of head and neck melanoma patients. Arch Otolaryngol Head Neck Surg.

[3] Grisham AD (2010). Giant melanoma: novel problem, same approach. South Med J.

[4] Panajotovic L, Dordevic B, Pavlovic MD (2007). A giant primary cutaneous melanoma of the scalp—can it be that big?. J Eur Acad Dermatol Venereol.

[5] del Boz J, Garcia JM, Martinez S, Gomez M (2009). Giant melanoma and depression. Am J Clin Dermatol.

[6] Tseng WW, Doyle JA, Maguiness S, Horvai AE, Kashani-Sabet M, Leong SP (2009). Giant cutaneous melanomas: evidence for primary tumour induced dormancy in metastatic sites?. BMJ Case Rep.

[7] De Giorgi V, Massi D, Carli P (2002). Giant melanoma displaying gross features reproducing parameters seen on dermoscopy. Dermatol Surg.

[8] Eisen DB, Lack EE, Boisvert M, Nigra TP (2002). Giant tumor of the back. Arch Dermatol.

[9] Kruijff S, Vink R, Klaase J (2011). Salvage surgery for a giant melanoma on the back. Rare Tumors.

[10] National Comprehensive Cancer Network (2011). NCCN Clinical Practice Guidelines in Oncology: Melanoma.

[11] Sloan AE, Nock CJ, Einstein DB (2009). Diagnosis and treatment of melanoma brain metastasis: a literature review. Cancer Control.

